# Beyond seizures: the hidden burden of developmental epileptic encephalopathies

**DOI:** 10.3389/fneur.2026.1960314

**Published:** 2026-10-14

**Authors:** Giovanni Battista Dell’Isola, Michele Sorrentino, Sara Massara, Gianluca Dini, Vincenzo Salpietro, Pietro Ferrara, Pasquale Striano, Federico Vigevano, Alberto Verrotti

**Affiliations:** 1 Department of Developmental Disabilities, IRCCS San Raffaele Roma, Rome, Italy; 2 Department of Medicine and Surgery, Saint Camillus International University of Health Sciences, Rome, Italy; 3 Department of Pediatrics, University of Perugia, Perugia, Italy; 4 Department of Biotechnological and Applied Clinical Sciences, Academic Unit of Pediatrics, University of L'Aquila, L'Aquila, Italy; 5 Department of Neuromuscular Diseases, Queen Square Institute of Neurology, University College London, London, United Kingdom; 6 Unit of Pediatrics, Campus Bio-Medico University, Rome, Italy; 7 Department of Neurosciences, Rehabilitation, Ophthalmology, Genetics, Maternal and Child Health, University of Genoa, Italy; 8 Pediatric Neurology and Muscular Diseases Unit, IRCCS Istituto “Giannina Gaslini”, Genoa, Italy

**Keywords:** developmental epileptic encephalopathy, Dravet syndrome, epilepsy, holistic approach, Lennox–Gastaut syndrome, tuberous sclerosis complex

## Abstract

Developmental and epileptic encephalopathies (DEEs) are a group of severe childhood-onset disorders characterized by drug-resistant epilepsy and a broad spectrum of neurodevelopmental, behavioral, psychiatric, and systemic manifestations. Although seizure control has traditionally been the primary therapeutic goal, growing evidence indicates that non-seizure manifestations often represent the main determinants of long-term disability, quality of life, and caregiver burden. This review highlights the hidden burden of DEEs by focusing on three paradigmatic syndromes: Lennox–Gastaut syndrome (LGS), Dravet syndrome (DS), and tuberous sclerosis complex (TSC). DS and LGS represent prototypical DEEs, whereas TSC is a multisystem genetic disorder that is frequently, although not invariably, associated with early-onset drug-resistant epilepsy and DEE-like neurodevelopmental trajectories. Across these conditions, the severity and progression of clinical manifestations are influenced by complex interactions between genetic, structural, developmental, and epileptic mechanisms, supporting the concept that epilepsy and associated manifestations represent interconnected features of a broader neurodevelopmental disorder. The review also emphasizes the substantial psychosocial, physical, and economic burden experienced by families and caregivers, which often exceeds the impact of seizures alone. Current management strategies should therefore extend beyond seizure reduction and adopt a multidisciplinary, life-course approach integrating early genetic diagnosis, syndrome-specific surveillance, developmental interventions, rehabilitation, behavioral support, and coordinated multidisciplinary care. Future research should prioritize disease-modifying therapies and personalized strategies aimed at addressing both epileptic and non-epileptic manifestations, ultimately improving functional outcomes and quality of life for individuals with DEEs and their families.

## 1 Introduction

The Developmental and Epileptic Encephalopathies (DEE) encompass a group of rare syndromes characterized by severe drug-resistant epilepsy with onset in the first years of life and significant neurodevelopmental disabilities. The phenotype is characterized by polymorphic seizures, intellectual disability, developmental and speech delay, behavioural problems, attention-deficit hyperactivity (ADHD) and autism spectrum disorders (ASD), psychiatric problems and sleep disorders. Epilepsy and marked epileptiform activity on EEG may contribute to or exacerbate the neurological condition (1, 2).

Traditionally, medical care of these syndromes has focused on controlling seizures. Nevertheless, there is an increasing recognition that non-epileptic features may have a greater impact on the burden of the disease compared to seizures. Etiological factors, including genetic, metabolic, and structural abnormalities, may play a major role not only in the development of epilepsy but also in the impairment of multiple domains of brain function (3). From an integrated, holistic perspective, epilepsy and associated neurodevelopmental disorders should therefore be considered interconnected manifestations, or coexpressions, of a broader underlying developmental brain disorder (4).

Dravet syndrome (DS), Lennox–Gastaut syndrome (LGS), and tuberous sclerosis complex (TSC) were selected as paradigmatic and complementary models to illustrate the heterogeneous relationship between epilepsy, neurodevelopmental impairment, and systemic disease burden. LGS represents a heterogeneous electroclinical DEE that may arise from multiple. DS represents a genetically defined DEE, most commonly related to SCN1A loss-of-function variants. TSC differs from DS and LGS because it is primarily a multisystem genetic disorder rather than invariably a DEE. However, TSC is frequently associated with early-onset drug-resistant epilepsy, developmental impairment, and TSC-associated neuropsychiatric disorders. For this reason, TSC provides a useful model to illustrate how epilepsy-related mechanisms, neurodevelopmental manifestations, and systemic complications may interact within a broader syndromic condition requiring lifelong surveillance and multidisciplinary management. Indeed despite a possible reduction of seizure frequency with age, the persistence of non-epileptic manifestations significantly affects the functional abilities, and quality of life of patients (5, 6). Genetic, structural, and developmental factors underlie both epileptic and non-epileptic manifestations, which proves the limited efficacy of a seizure-centered approach and the need for comprehensive, multidisciplinary care approaches.

The purpose of this review is therefore not to provide an exhaustive overview of all DEEs, but to use these three conditions as representative examples of different but overlapping clinical scenarios: a monogenic channelopathy, a heterogeneous electroclinical syndrome, and a multisystem genetic disorder frequently associated with DEE-like trajectories. By comparing these conditions, we aim toto summarize the current knowledge on the broader disease burden of DEE and to emphasize the need for holistic treatment approaches.

## 2 Lennox–Gastaut syndrome

LGS is a rare and severe form of childhood-onset epilepsy, that affects around 1–2% of all epilepsy patients. LGS is characterized by multiple seizure types, which frequently are not responsive to treatment.

LGS arises from a broad range of etiologies, resulting in marked heterogeneity of neurological manifestations. Consequently, the presence and severity of non-seizure manifestations vary according to the underlying pathogenesis (Figure 1).

**Figure 1:**
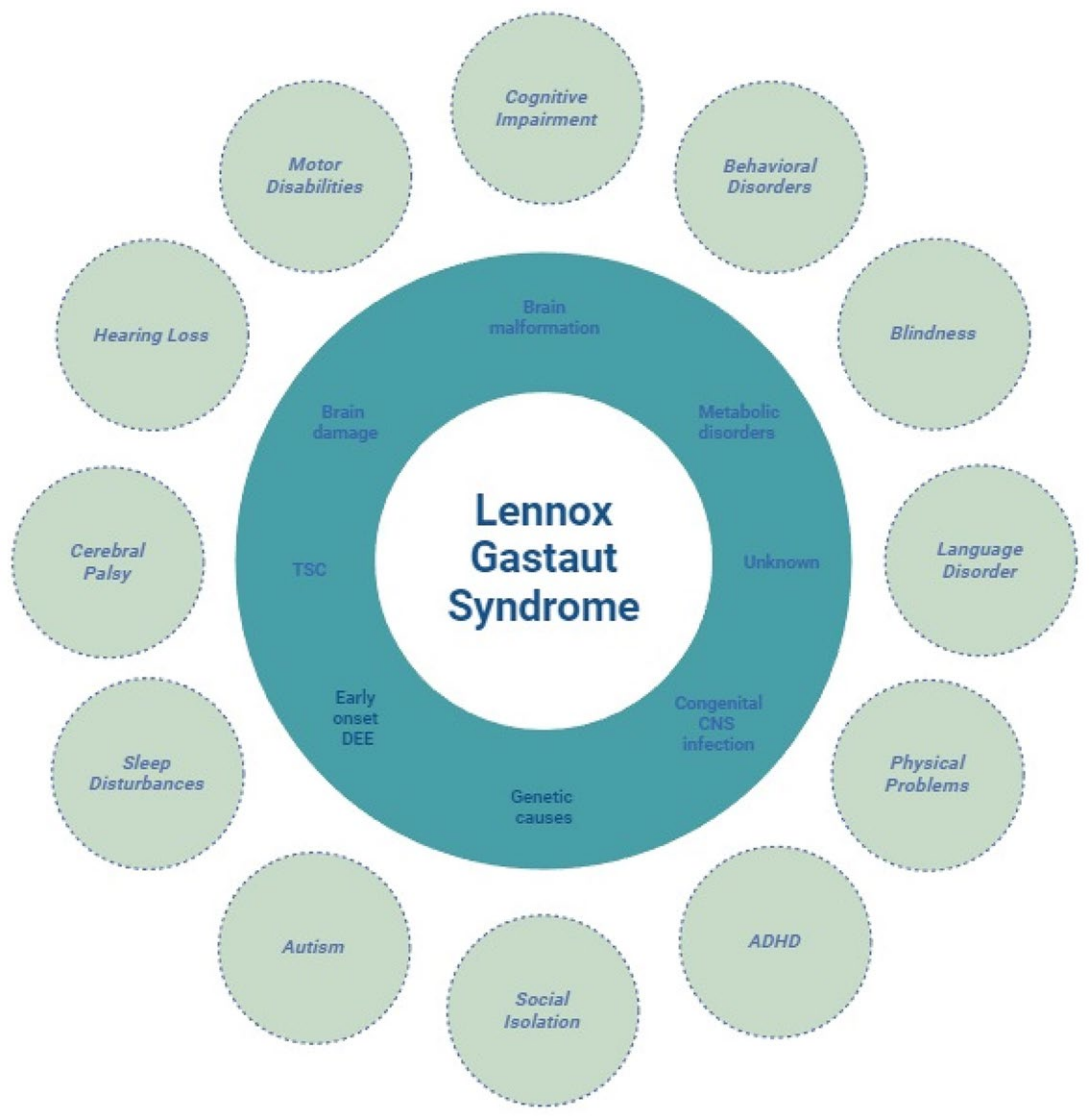
The inner circle depicts the various etiologies underlying LGS. The outer circles illustrate the spectrum of non-epileptic comorbidities associated with LGS, which are influenced by the specific underlying etiology.

Beyond epilepsy, LGS is associated with numerous neurological manifestations, among which developmental delay is particularly prominent. Indeed, developmental delay constitutes one of the three core diagnostic criteria for LGS, together with the presence of multiple seizure types and a characteristic electroencephalographic pattern (7). At the time of diagnosis, developmental delay is observed in 20–60% of patients, particularly in secondary forms of LGS. Approximately 30% of cryptogenic cases develop developmental delay only after seizure onset.

Approximately 75–95% of individuals with LGS develop cognitive impairment within five years of disease onset (8), ultimately resulting in moderate to severe intellectual disability in more than 90% of patients over time (9). Age at onset shows a strong association with clinical presentation (10). In particular, early-onset epilepsy and LGS evolving from infantile epileptic spasm syndrome are associated with a more severe impact on cognitive development, reflecting the effects of epileptic encephalopathy during critical periods of brain maturation (11).

Motor impairments, including ataxia, spasticity, and extrapyramidal signs, are frequently observed in individuals with LGS (12). In a cohort study of 26 patients with LGS (mean age 16.6 years), substantial motor involvement was reported, with 43% of patients dependent on mobility aids and only 19% exhibiting preserved gross motor function (13).

In the initial phase of the disease, the patients commonly present hyperkinesia and apraxia, which result in great problems with performing coordinated and goal-oriented movements (11). As disease progresses, ideomotor slowing may become so pronounced that it hides any hyperkinetic or perseverative behaviors (11).

Behavioral problems are also common in LGS, occurring in about 50% of individuals. These manifestations include hyperactivity, aggression, and autistic traits (9, 14).

Sleep disorders represent another characteristic feature. Tonic seizures during sleep frequently occur in LGS patients and may profoundly disrupt sleep architecture (9). Besides, the use of polypharmacotherapy and various environmental factors, contribute to a higher prevalence of sleep problems, including insomnia, and excessive daytime sleepiness (15).

However, apart from the neuropsychiatric manifestations, most LGS patients usually suffer from systemic medical problems related to the underlying etiology. Such problems include urinary incontinence, osteoporosis, and gastrointestinal complaints. Renal and lung pathology is especially common for LGS associated with TSC (12).

The presence of comorbidities significantly impacts the quality of life of both patients and caregivers. Developmental delay and intellectual disability are nearly always present in LGS and represent major contributors to communication difficulties. Speech disorders are reported in 40–96% of cases (16), with many patients remaining nonverbal. These impairments severely limit peer interactions (13) and significantly impacts family dynamic. It becomes difficult for caregivers to understand the needs of individuals who mostly rely on nonverbal communication strategies such as gestures, facial expression, gaze, and vocalizations (13).

Motor impairment combined with frequent seizures, especially drop attacks, contribute to substantial physical morbidity. Frequent falls, poor walking abilities, and the necessity for protective equipment, such as helmets, are common in people with LGS. Limited mobility and sedentary behavior further increase the risk of obesity, osteoporosis, and fractures (16).

The physical fragility of patients with LGS makes them vulnerable to infections, particularly respiratory infections, that lead to hospitalization and respiratory support (17).

Loss of independence affects even basic daily activities. Around 30% of patients need enteral feeding (13), and personal hygiene mostly relies on caregivers (16). Such a restriction results in a vicious cycle where behavioral disorders are enhanced, depending on the degree of cognitive impairment and awareness of the condition. Furthermore, behavioral issues prevent patients from taking part in social life (9). Only a small minority of individuals with LGS can manage to learn to drive, and most adults remain illiterate being forced to reside in long-term care facilities (16).

Moreover, pharmacological therapy increase the disease burden. Using polytherapy in the treatment of seizures and associated manifestations raises the risk of drug side effect and may worsen emotional and behavioral disorders.

The overall effect of comorbid conditions associated with LGS imposes a substantial economic burden on families, who often incur significant costs related to assistive devices, specialized treatment, and long-term care.

## 3 Dravet syndrome

DS is a severe DEE, generally caused by heterozygous loss-of-function variants in *SCN1A*, which encodes the voltage-gated sodium-channel α-subunit Nav1.1. The severity of DS varies widely both in seizure burden and associated manifestations (18). Developmental milestones are generally on track during the first year of life, although school-aged individuals with DS show varying levels of developmental delays that could influence learning, cognitive abilities, language, and motor development. Adolescents and adults with DS are usually affected by moderate to severe intellectual disability (19). While DS is a result of specific genetic abnormalities, other possible contributors of developmental delays include frequent occurrence of seizure, drug side effects, and the administration of contraindicated drugs (20).

Autistic symptoms, Attention Deficit Disorder or ADHD are prevalent in DS (21). Irritability and aggressive behavior are also common and, while challenging at all ages, these behaviors can become particularly difficult to manage as children grow into adulthood (22). Unlike other traits shared with ASD, patients with DS are often overly social, although some may prefer interactions with adults over peers. Depressive and anxiety symptoms have been reported, particularly during adolescence and adulthood.

Sleep disturbances are a significant issue in DS. Children may have difficulty maintaining regular sleep patterns, which can affect the whole family. In addition, increased seizure activity during sleep may occur. Seizure monitoring is critical considering the increased risk of status epilepticus and sudden unexpected death in epilepsy (SUDEP) in DS patients (23).

Movement, balance, and gait problems, along with low muscle tone can appear with growth (24). Adults with DS frequently have a crouched gait and may require mobility assistance (25). However, the progression of motor impairment is not fully understood, and no expert recommendations exist regarding treatment options (26).

There is limited research on cardiac functioning in DS. However, some evidence indicates that alterations in heart rate and/or cardiac rhythm may occur. The *SCN1A* gene is also expressed in cardiac cells, which may explain these risks. As a consequence, the increased risk of SUDEP in DS may be partly related to subtle cardiac abnormalities (27).

The prevalence of dysautonomia in DS is not well understood (13). However, since seizure may be triggered by changes in body temperature, families often take extra precautions, such as avoiding extreme water temperature or controlling home environment.

Besides the phenotypic characteristics related to the *SCN1A* gene variant, the magnitude of cognitive, motor, and behavioural impairment is influenced by seizure frequency and disease course (28). Some of the disorders are likely to be directly caused by *SCN1A* gene variants (29), for instance, ataxia and gait disturbances, while some others may appear as sequels of the epileptic encephalopathy. Patients with higher seizure burden are characterized by more severe motor and language skills impairment. However, the causal relationships between seizures and non-epileptic manifestations remain difficult to separate based on observational data alone. By contrast, behavioral and neurodevelopmental disorders, such as ASD, ADHD, and other behavioral problems, do not consistently correlate with seizure frequency.

## 4 Tuberous sclerosis complex

TSC is an inherited condition caused by pathogenic variants in either the *TSC1* or *TSC2* genes, which lead to the dysregulation of the mechanistic target of rapamycin (mTOR) signaling pathway. This alteration results in the formation of benign tumors in various organ systems, leading to a wide array of comorbid conditions that significantly impact the clinical course of TSC (30).

Beyond epilepsy, TSC is associated with a wide range of neuropsychiatric manifestations known collectively as TSC-associated neuropsychiatric disorders (TAND) (31). These include cognitive impairment, ASD, and behavioral disturbances. Systematic TAND screening across the lifespan is therefore essential, as it allows cognitive, behavioral, psychiatric, academic, and psychosocial manifestations to be identified and monitored within a structured multidimensional framework rather than as isolated complications.

Cognitive functioning in TSC ranges from normal intellectual functioning to severe intellectual disability, with intellectual disability reported in approximately 53.6–65% of patients. Cognitive impairment is influenced by several factors such as the size and location of cortical tubers, and seizure management (31).

Intellectual disability, developmental delay, and early-onset drug-resistant epilepsy represent major risk factors for the occurrence of ASD among patients with TSC (32), with prevalence rates ranging from 30 to 60% (33). The pathogenic variants in *TSC2* are more frequently associated with both epilepsy and ASD, suggesting a genotype–phenotype correlation (34).

ADHD is more common during childhood, affecting approximately 25% of pediatric patients. Anxiety and depression are more prevalent among adults, with a 15–20% prevalence rate (35).

Sleep disturbances are highly prevalent in individuals with TSC and are strongly associated with TAND, affecting up to 60% of patients (36). Disturbances of sleep–wake transitions (26.5%), difficulties in falling and staying asleep (22%), and sleep-related breathing disorders (19.2%) are the sleep disorders most frequently observed (36).

Renal involvement represents another hallmark of TSC. Renal angiomyolipomas (AMLs) and renal cysts are the most common renal manifestations, occurring in almost 50% of individuals (37, 38). Although AMLs are benign tumors, they may result in significant morbidity, including hemorrhage, hypertension, and kidney failure (39).

Cutaneous involvement is quite distinctive of TSC and serves as a useful diagnostic marker. This includes hypomelanotic macules (66.7–97.2%), facial angiofibromas (57.3–74.5%), and shagreen patches (22.7–48.1%). Other skin lesions that occur less frequently include molluscum fibrosum pendulans (22.6%), forehead plaques (18.9%), and periungual fibromas (15.1%) (40).

Cardiac rhabdomyomas, frequently detected prenatally or in early infancy, often represent the earliest manifestation of TSC. These tumors typically regress spontaneously. However, they may occasionally lead to significant complications, such as arrhythmias, heart failure, or obstruction of intracardiac blood flow (41).

Pulmonary involvement in TSC primarily manifests as lymphangioleiomyomatosis (LAM), a progressive cystic lung disease affecting approximately 30% of individuals with TSC. LAM mainly occurs in women of reproductive age and may lead to a progressive decline of pulmonary function (42).

TSC may also involve other organs. Ocular manifestations include retinal hamartomas, occurring in 30–44% of individuals, and chorioretinal hypopigmentation, in approximately 39% of patients (43). Less frequent systemic manifestations include hepatic AMLs and hepatic cysts, reported in approximately 9.1% of cases (44), and pancreatic neuroendocrine tumors, which have been described in about 4.1% of individuals with TSC (45).

In order to properly assess the burden of TSC-associated comorbidities, it is relevant to consider not only their prevalence but also the natural history. Comorbidities do not arise simultaneously and often vary across the lifespan (Figure 2). The need for lifelong surveillance across multiple organ systems represents an additional component of the disease burden in TSC (Table 1). Furthermore, the impact on the quality of life is not equal throughout life. Dermatologic manifestations provide a clear example of this age-dependent effect. Although these lesions are generally benign, they usually gain higher psychological and social significance during the adolescence and adulthood when cosmetic concerns become prominent.

**Figure 2:**
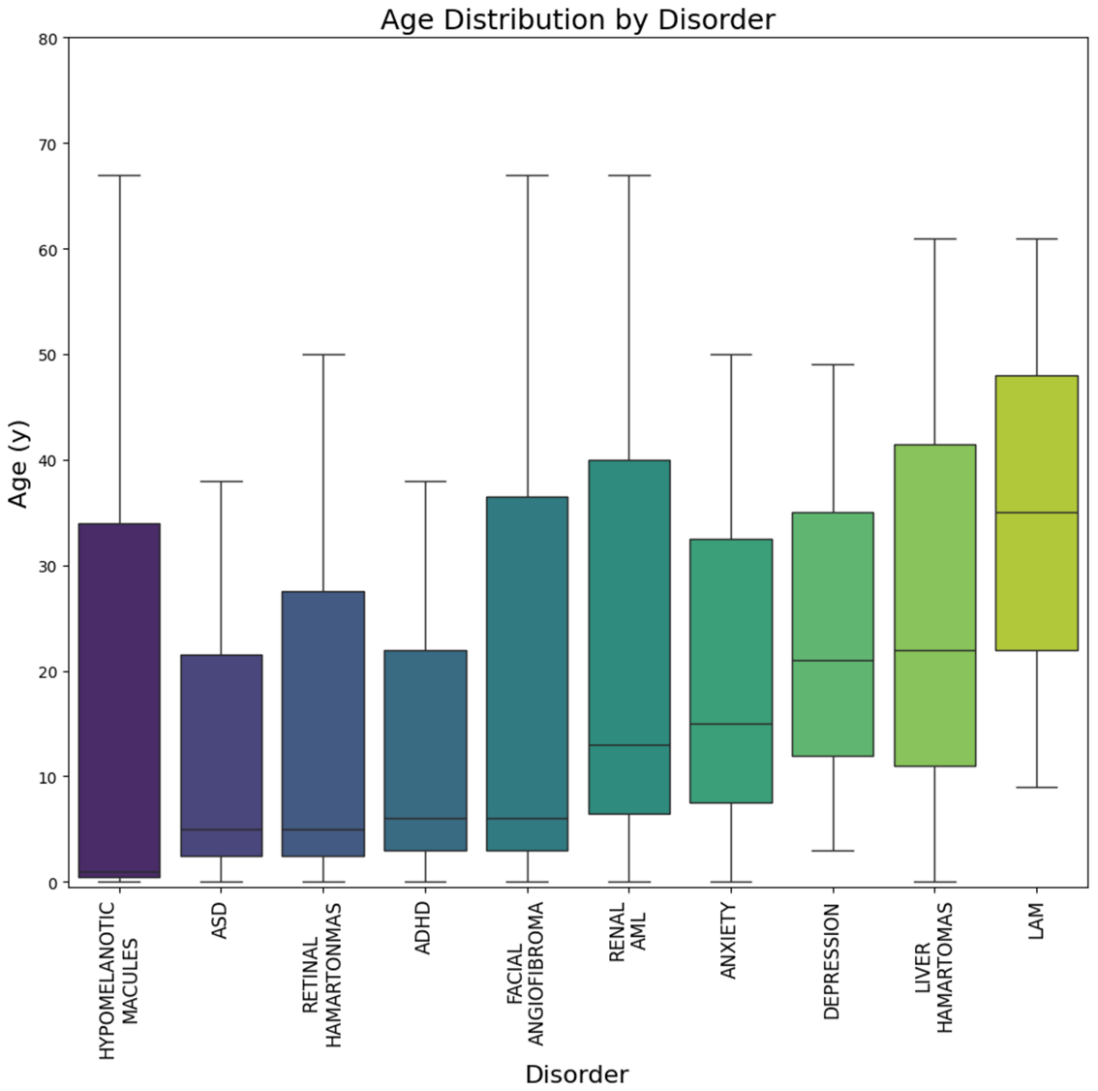
Age distribution of TSC-associated disorders. Data from: Kingswood et al. (37).

**Table 1:** Timing of surveillance for non-epileptic manifestations in TSC. Data from: Northrup et al. (39).

System	Timing of surveillance recommendations
Brain	MRI every 1–3 years in asymptomatic patients or more frequently in case of complicated SEGA. EEG should be performed every 6 weeks until 12 months of age and every 3 months until 24 months of age. Subsequent timing should be based on the characteristics of neuropsychiatric disordersx. At least one annual evaluation of TSC-associated neuropsychiatric disorders should be conducted.
Kidney	MRI every 1–3 years. Blood tests to assess renal function should be performed at least annually.
Lung	Chest CT should be conducted every 5–7 years until menopause. The timing of chest CT scans should be individualized based on the specific needs and progression of LAM in affected patients. At least annual pulmonary function testing should be conducted to monitor patients with LAM.
Skin	Annual dermatological evaluation.
Teeth	Dental examination at least every 6 months.
Heart	Echocardiography should be performed every 1–3 years until regression of cardiac rhabdomyomas. Evaluations may be more frequent in cases of symptomatic rhabdomyomas. ECG every 3 to 5 years.
Eye	Annual ophthalmic evaluation.

Compared with DS and LGS, TSC is associated with a lower prevalence of intellectual disability. This relative preservation of cognitive function could results in increased awareness of the disease and heighten the effects of comorbidities on patients quality of life and their interactions in society. Individuals with TSC live with the persistent risk of lesion growth and possible malignant transformation. This uncertainty creates a continuous psychological stress and partly explain the high prevalence of anxiety and depression. Indeed, mood disorders are more prevalent in adults with TSC patients (35).

## 5 Integrated management and therapeutic priorities

Given the broad spectrum of neurodevelopmental, behavioral, motor, sleep-related, autonomic, and systemic manifestations, the management of DEE requires an integrated approach that extends beyond seizure control. Although seizure reduction remains a fundamental therapeutic goal, a seizure-centered strategy risks underestimating the overall disease burden and the well-being of both patients and families. Therefore, therapeutic decisions should be guided by a global assessment of the patient, taking into account the full clinical picture rather than focusing exclusively on seizure frequency. Management should be patient-centered and goal-oriented, recognizing that meaningful improvement may involve communication, sleep, mobility, behavior, autonomy, participation, or family functioning.

In this context, a major limitation lies in the difficulty of assessing non-seizure outcomes. Unlike seizure frequency, which can be quantified through seizure diaries, EEG recordings, or event monitoring, neurodevelopmental, behavioral, motor, sleep-related, autonomic, systemic, adaptive-functioning, and quality-of-life outcomes are often more difficult to measure objectively. This is particularly relevant in individuals with severe developmental impairment, limited or absent expressive language, behavioral dysregulation, and high dependence on caregivers, in whom subjective symptoms such as pain, distress, sleep quality, mood, fatigue, or discomfort may be difficult to identify and may instead manifest through changes in behavior, alertness, feeding, sleep, or participation in daily activities. As a consequence, the evaluation of non-seizure manifestations often requires a multidimensional approach combining structured clinical assessments, functional scales, neuropsychological and behavioral measures when feasible, and longitudinal observation across developmental stages. The lack of standardized, sensitive, and syndrome-specific tools for measuring these outcomes remains a major limitation in therapeutic trials.

The perspectives of patients and caregivers should therefore be explicitly incorporated into this process. Patient- and caregiver-reported outcomes should be integrated with clinical and functional assessments to identify treatment goals that are meaningful in everyday life and to evaluate therapeutic benefits beyond seizure frequency.

Early diagnosis represents a key therapeutic priority, especially in genetically defined syndromes such as DS and TSC. Advances in genetic testing have shortened diagnostic delays, allowing earlier access to syndrome-specific treatments, avoidance of inappropriate drugs, and timely referral to developmental interventions. Early intervention, special educational support, physiotherapy, occupational therapy, and speech therapy should be tailored to the patient developmental needs, with the aim of maximizing functional abilities and long-term outcomes (46). The identification of clinical, electroencephalographic, neuroimaging, genetic, and serum biomarkers associated with ASD may support earlier and more targeted interventions (32, 47), although the impact of presymptomatic epilepsy treatment on cognitive and neuropsychiatric outcomes remains controversial (48, 49).

Non-pharmacological and supportive interventions are essential components of care. Rehabilitation, speech and augmentative communication strategies, nutritional support, pain assessment, behavioral and educational interventions, and caregiver support should be integrated into routine clinical practice.

Motor and gait abnormalities require regular functional assessment and, when appropriate, physiotherapy, occupational therapy, orthotic devices, mobility aids, and individualized strategies to preserve autonomy. Sleep disturbances should be actively investigated and managed, as they affect both patients and families and may interact with nocturnal seizures and seizure-related risks.

TSC represents a paradigmatic example of the need for syndrome-specific surveillance and targeted therapy. Due to TSC multisystem involvement, long-term management requires periodic monitoring of neurological, renal, pulmonary, dermatological, cardiac, dental, and ophthalmological manifestations. Advances in the understanding of mTOR dysregulation have enabled the development of targeted treatments, including everolimus and sirolimus, which have demonstrated efficacy in reducing renal angiomyolipoma size, stabilizing renal function, and improving seizure control (50–52). However, despite possible disease-modifying effects (53), its impact on neurocognitive and behavioral outcomes remains inconclusive (54). Moreover, the use of mTOR inhibitors requires careful long-term monitoring because of immunosuppressive effects and increased susceptibility to infections (50).

Overall, these considerations support the need for individualized, multidisciplinary, and life-course care models according to the syndrome and the patient’s clinical needs. Structured tools and timelines for monitoring neuropsychiatric progression may help optimize follow-up and guide timely interventions (15). Future research should prioritize disease-modifying therapies and clarify the mechanisms underlying neurodevelopmental trajectories beyond seizure burden alone. In particular, a better understanding of genotype–phenotype correlations, epileptic and non-epileptic disease mechanisms, and the long-term effects of early targeted interventions may support more personalized strategies aimed at reducing the burden of comorbidities and improving functional outcomes and quality of life.

## 6 Discussion

Historically, epilepsy and its associated conditions have been conceptualized as distinct yet coexisting entities. However, the advances in the understanding of epilepsy syndromes, particularly DEE, have increasingly challenged paradigm. There is growing evidence that the non-epilepsy signs and symptoms associated with DEEs and other epilepsy syndromes are integral parts of the disease that play important roles in diagnosis, therapeutic choice, and prognosis.

A central challenge in the DEE concept is to define the relative contribution of the underlying developmental disorder and epileptic activity to the overall phenotype. In many cases, cognitive, behavioral, motor, and sleep-related manifestations are likely to reflect shared genetic, structural, and developmental mechanisms, while seizures and interictal epileptiform activity may further modify or amplify these trajectories during sensitive periods of brain maturation. Thus, the relationship between epilepsy and non-seizure manifestations should not be interpreted as strictly causal or unidirectional, but rather as dynamic and interdependent.

This prospective has prompted a paradigm shift from the concept of “comorbidity” toward a more holistic approach, in which epilepsy represents one of multiple interrelated feature of a broader neurodevelopmental disorder.

In this context, syndromes such as DS and LGS exemplify the dynamic natural history of DEEs. As patients age from childhood to adolescence and adulthood, seizure frequency may decreases (16). Consequently, the role of seizures in the burden of DEEs decreases in importance, and it becomes secondary to other symptoms, including cognitive impairment, behavior problems, movement disorders, sleep problems, and systemic complications. Across LGS, DS, and TSC, cognitive impairment, behavioral manifestations, sleep disturbances, motor dysfunction, reduced autonomy, and caregiver burden emerge as recurrent non-seizure determinants of disease burden, although their relative contribution and clinical expression vary according to the underlying syndrome.

The problem is especially clear for LGS, as intellectual disability, loss of autonomy, and behavioral dysregulation affect daily functioning more than seizure (9, 16). Likewise, in TSC, neuropsychiatric symptoms, progression to affect multiple systems, and lifelong monitoring and interventions become more significant than the direct effect of seizures on the patients lives (55). Sleep disturbances further illustrate why disease burden cannot be captured by seizure frequency alone, as they may interact bidirectionally with nocturnal epileptic activity, ASM, daytime alertness, behavioral regulation, and caregiver burden.

Recognizing the importance of these manifestations also requires improving how they are measured. The predominance of seizure frequency as an outcome in clinical practice and therapeutic trials partly reflects its relative ease of quantification, whereas non-seizure outcomes are multidimensional, fluctuate across development, and often rely on caregiver observation, particularly in individuals with severe intellectual disability or limited communication abilities.

Of significance, the disease burden extends beyond the affected individual. There is considerable psychological, physical, and financial stress on families and caregivers, resulting from the burden of caring for patients, loss of autonomy, constant admissions to the hospitals, and need for special interventions. Often specialized tertiary care centers that may be geographically distant from patients homes are involved. This imposes substantial direct and indirect costs and contributes to a significant psychological burden on patients and their families. Diagnostic delays and fragmented care, particularly during the transition from pediatric to adult services, further exacerbate this burden (56).

A patient- and caregiver-centered perspective is therefore essential to define clinically meaningful outcomes in DEEs. Incorporating these perspectives is critical for aligning therapeutic strategies with real-life needs and for designing trials that capture outcomes that matter to patients and caregivers.

The growing availability of targeted therapies, such as mTOR inhibitors in TSC, represents a major advance. However, current evidence suggests that seizure control and modulation of single pathogenic pathways are insufficient to modify neuropsychiatric outcomes in patients with DEE (48, 49). These observations point to the role of additional mechanisms that are responsible for the development of the diverse phenotypic picture of DEE.

## 7 Conclusion

While epilepsy may be considered an important feature of DEE, it does not play the only role in determining the burden of disease. Non-epileptic features of syndromes like DS, LGS, and TSC are prevalent and multi-faceted, being even more burdensome than seizures.

Since seizure burden may reduce with time, non-epileptic issues gain even more importance. They affect people ability to live independently and participate socially as well as impose a heavy burden on the families and caregivers (16).

The future advances in the treatment of DEE are likely to be linked to a paradigm shift towards holistic approaches to care, which include not only seizure control but also early detection and management of non-epileptic manifestations.

Simultaneously, scientific investigations should focus on disease-modifying therapy with the aim of changing the course of global neurodevelopmental trajectory, particularly in condition where initial impairment is less prominent.
